# Increased serum piwi-interacting RNAs as a novel potential diagnostic tool for brucellosis

**DOI:** 10.3389/fcimb.2022.992775

**Published:** 2022-09-15

**Authors:** Cheng Wang, Cuiping Zhang, Quan Fu, Nan Zhang, Meng Ding, Zhen Zhou, Xi Chen, Fengmin Zhang, Chunni Zhang, Chen-Yu Zhang, Jun-Jun Wang

**Affiliations:** ^1^ Department of Clinical Laboratory, Jinling Hospital, State Key Laboratory of Analytical Chemistry for Life Science, NJU Advanced Institute for Life Sciences (NAILS), School of Life Sciences, Nanjing University, Nanjing, China; ^2^ Nanjing Drum Tower Hospital Center of Molecular Diagnostic and Therapy, Chinese Academy of Medical Sciences Research Unit of Extracellular RNA, State Key Laboratory of Pharmaceutical Biotechnology, Jiangsu Engineering Research Center for MicroRNA Biology and Biotechnology, NJU Advanced Institute of Life Sciences (NAILS), Institute of Artificial Intelligence Biomedicine, School of Life Sciences, Nanjing University, Nanjing, China; ^3^ Department of Prenatal Diagnosis, Women’s Hospital of Nanjing Medical University, Nanjing Maternity and Child Health Care Hospital, Nanjing, China; ^4^ Department of Microbiology, Harbin Medical University, Harbin, China; ^5^ Department of Clinical Laboratory, Affiliated Hospital of Inner Mongolia Medical University, Hohhot, China

**Keywords:** piRNA, serum, brucellosis, biomarker, qRT-PCR

## Abstract

**Background:**

Piwi-interacting RNAs (piRNAs) have emerged as potential novel indicators for various diseases; however, their diagnostic value for brucellosis remains unclear. This study aimed to evaluate the diagnostic potential of altered serum piRNAs in patients with brucellosis.

**Methods:**

Illumina sequencing *via* synthesis (SBS) technology was used to screen the serum piRNA profile in brucellosis patients, and markedly dysregulated piRNAs were confirmed by quantitative real-time polymerase chain reaction (qRT-PCR) assay in two sets from a cohort of 73 brucellosis patients and 65 controls.

**Results:**

Illumina SBS technology results showed that seven piRNAs were markedly elevated in brucellosis patients compared to normal controls. The seven upregulated piRNAs were further validated individually by qRT-PCR, of which three piRNAs (piR-000753, piR-001312, and piR-016742) were confirmed to be significantly and steadily increased in the patients (> 2-fold, P < 0.01). The area under the receiver operating characteristic (ROC) curve (AUCs) for the three piRNAs ranged from 0.698 to 0.783. The AUC for the three piRNAs combination was 0.772, with a specificity of 86% and a positive predictive value of 90%, respectively.

**Conclusions:**

The three-piRNA panel identified in this study has potential as a novel blood-based auxiliary tool for brucellosis detection.

## Introduction

Brucella is a facultative intracellular gram-negative germ that can induce a zoonotic bacterial disease called brucellosis in humans and animals (Zai et al., 2017). Brucellosis primarily affects the reproductive tract, resulting in abortion and infertility in the natural host (Ahmed et al., 2016). In addition, Brucella is responsible for severely debilitating and disabling illnesses, such as fever, chills, sweats, weakness, and some complications (Rossetti et al., 2017). There are 12 Brucella species based on their unique association with a natural host; however, only B. melitensis, B. abortus, B. suis, and B. canis are related to human diseases (Hull and Schumaker, 2018). It has been reported that the number of people with brucellosis is 500,000 annually; however, it appears that the actual number is far greater than that (Marvi et al., 2018). Humans are infected with Brucella mainly by direct or indirect contact with infected animals or contaminated animal products, and the main source of infection is livestock such as sheep, goats, buffaloes, and steers (Budak et al., 2016; Budak et al., 2016). In China, B. melitensis accounts for most brucellosis cases in humans, and high-prevalence areas are mainly concentrated in Inner Mongolia, Xinjiang, Qinghai, Ningxia, and Henan provinces (Lai et al., 2017). In epidemic areas, brucellosis causes severe economic damage and raises public health risks. However, this disease lacks specific clinical manifestations and is difficult to distinguish without laboratory examination (Andriopoulos et al., 2007). At present, major laboratory tests include blood culture, serological testing, and molecular detection. Blood culture is the gold standard for laboratory confirmation; however, its sensitivity depends on the stage of brucellosis and previous use of antibiotics. In addition, a prolonged incubation time of up to 7 days and a low positive rate limit its application. Serological testing mainly involves agglutination tests and ELISA, which still have limitations due to the high background values in endemic areas and the cross-reactivity of smooth lipopolysaccharide antigen with other gram-negative bacteria (Zerva et al., 2001; Franco et al., 2007; Al Dahouk et al., 2013; Wareth et al., 2016; Hull et al., 2018). Therefore, further exploration is urgently required to improve the diagnosis of brucellosis.

Piwi-interacting RNAs (piRNAs), a subset of small non-coding RNAs with a length of 23–36 nucleotides, interact with PIWI proteins in germ and stem cells to silence transposable elements in the genome at the transcriptional level and are involved in the pathophysiological processes of various diseases (Sato and Siomi, 2013; Assumpcao et al., 2015; Iliev et al., 2016; Kamaliyan et al., 2018; Roy and Mallick, 2018; Zhang et al., 2018). Specific piRNAs in circulation or other body fluids have been considered potential biomarkers for various diseases (Cheng et al., 2011; Iliev et al., 2016; Weng et al., 2018; Vychytilova-Faltejskova et al., 2018; Wang et al., 2020; Ge et al., 2020; Li et al., 2021; Jia et al., 2021). Our previous study demonstrated a distinctive panel of piRNAs in seminal plasma that could accurately distinguish between infertile and fertile males and might serve as a promising molecular indicator of male infertility (Hong et al., 2016). We suspected that patients with Brucella infection may present clinically significant differences in the circulating levels of piRNAs. Therefore, this study aimed to investigate the differentially expressed piRNAs in the sera of brucellosis patients and normal controls, and to further explore their clinical significance.

## Materials and methods

### Enrollment of patients and controls

The study cohort comprised 73 brucellosis patients recruited at the Affiliated Hospital of Inner Mongolia Medical University or Zhungeer Qi Center for Disease Control and Prevention, Inner Mongolia, China, from 2016 to 2017. The brucellosis group included 48 males and 25 females, and the mean of their age was 47.55 ± 12.88 years and 45.51 ± 9.01 years, respectively. All patients underwent routine laboratory tests. Blood cultures were positive in 18 (25%) patients, and the others were diagnosed using serological tests. Sixty-four (88%) patients had underlying acute infections, whereas the others had chronic infections. Most of the patients (68/73, 94%) in our study were Han Chinese. In addition, 64 patients (87.5%) were newly recruited without any treatment, and only nine patients (12.5%) were undergoing anti-bacterial treatment. At the same time, we recruited 65 healthy Han Chinese matched for age and sex as the parallel control group from a large pool of individuals seeking routine health checkups at the Healthy Physical Examination Center of Jinling Hospital, Nanjing, China. The selected controls underwent routine laboratory and imaging tests and exhibited no clinical symptoms of Brucella infection. The indicators of all the control individuals were checked normally and showed no evidence of disease. The control group contained 41 males and 24 females, with an average age of 48.00 ± 14.18 years and 42.46 ± 16.62 years, respectively. There was no statistical difference in the distribution of age and sex between the patient and control groups (age, P = 0.172; sex, P = 0.743).

This study was approved by the ethics committee board of each sample collection institution, and written informed consent was obtained from all participants. This study complied with the principles of the World Medical Association Declaration of Helsinki.

### Sample preparation

Blood samples collected from each participant were compliant with standard operating procedures as previously described (29). All samples were collected in gel separator/coagulant tubes and centrifuged at 1500 × g for 10 min at room temperature. Thereafter, the supernatants were centrifuged at 2000 × g for 10 min at 4 °C to remove debris. The serum supernatant samples were stored at -80 °C until further processing.

### Study design

**Figure 1:**
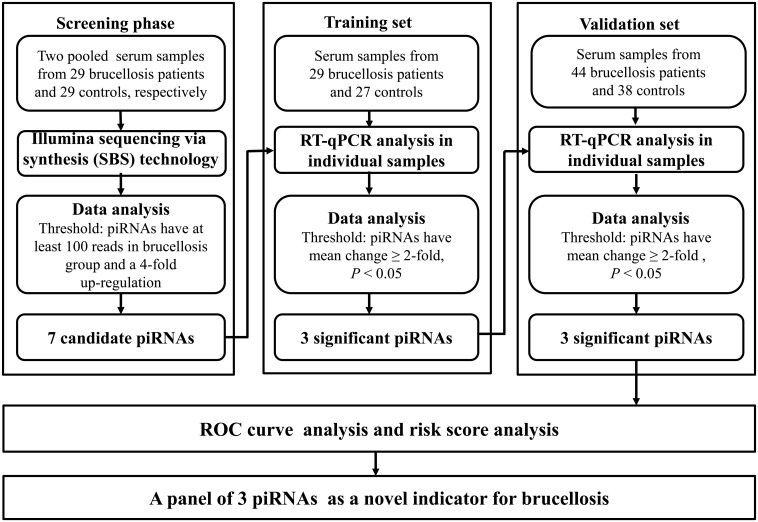
Flow chart of the experimental design.

A multiphase retrospective case-control study was conducted to identify significantly and steadily upregulated serum piRNAs as successful blood examination indicators for the diagnosis of brucellosis (**Figure 1**). In the initial screening phase, we prepared two pools of serum samples from 29 patients with brucellosis and 29 healthy controls. RNA was isolated from the two pools, which was then applied to Illumina SBS technology to identify piRNAs that showed distinct alterations between the two groups. Thereafter, confirmation analyses were performed using qRT-PCR in two sets to refine the altered serum piRNAs selected from the initial screening phase: (a) training set, in which serum samples were composed of 29 brucellosis patients and 27 control individuals, including samples from the screening phase, and (b) validation set, which consisted of an additional 44 patients with Brucella infection and 38 normal controls.

### RNA extraction and Illumina SBS technology

RNA extraction for Illumina SBS technology and qRT-PCR assays was performed as previously described (Zhang et al., 2010; Yan et al., 2015). Illumina SBS technology (BGI Genomics Co., Shenzhen, China) was used to separately isolate RNA from two pooled samples, as previously described (Ding et al., 2018). Further details are shown in **Supplementary Materials**.

### Assay precision of qRT-PCR for piRNA

Since only a few reports and no standard method focused on body fluid piRNAs study currently, the repeatability of serum RNA extraction, the analytical repeatability of the qRT-PCR assay, and the assay precision of the piRNAs quantification were conducted to evaluate the serum piRNA detection system. Further details are shown in the **Supplementary Materials**.

### Quantification of piRNA by qRT-PCR analysis

In this study, qRT-PCR was performed following the manufacturer’s instructions (Roche Light Cycler® 480 II, Roche Diagnostics Ltd., Rotkreuz, Switzerland) to validate the differentially expressed piRNAs between brucellosis patients and normal controls, as described previously (Hong et al., 2016).

To control for variability in RNA extraction and purification procedures, we added an exogenous reference gene, plant miRNA MIR2911 (5′-GGCCGGGGGACGGGCUGGGA-3′), to each serum sample at a final concentration of 10^6^ fmol/L during RNA isolation, as previously reported with minor modifications (Yan et al., 2015). For the Cq values of MIR2911, no significant difference was observed between the patient and control groups (P > 0.05) (**Supplementary Figure 1**). The relative contents of serum piRNAs were normalized to MIR2911 and calculated using the 2^−ΔCq^ method, in which ΔCq = Cq _[target piRNA]_ – Cq _[MIR2911]_.

### Statistical analysis

All statistical analyses were performed using the SPSS software (version 23.0; IBM, NY, USA) and GraphPad Prism 6 (GraphPad Software, CA, USA). Data are presented as the mean ± SEM for piRNAs. The nonparametric Mann–Whitney U test was used to compare differences in variables between the groups. We established receiver operating characteristic (ROC) curves and calculated the areas under the ROC curve (AUC) to evaluate the specificity and sensitivity of candidate piRNAs for brucellosis diagnosis. The positive predictive value (PPV) and negative predictive value (NPV) were calculated to determine the diagnostic ability for brucellosis. Risk score analysis was performed to assess the associations between the combination of piRNAs and brucellosis, as previously described (Zhang et al., 2010). Statistical significance was set at P < 0.05.

## Results

### Serum piRNA profile analyzed by Illumina SBS technology

We used Illumina SBS technology to initially search for differentially expressed serum piRNAs in patients with brucellosis compared to normal controls. Unique sequences with lengths of 17–44 nucleotides were first mapped to the Brucella genome, and the unmapped sequences were then aligned to human genomes. The mapped reads were used for further analyses. These sequences were annotated using the miRBase, tRNA, rRNA, and rfam databases. The unmapped reads were finally mapped to piRNABank, and 25838 piRNAs, including 708 known and 25130 novel piRNAs, were detected. Of the 708 known piRNAs that were annotated in the NCBI database, 646 piRNAs showed more than 2-fold change between the patients and the controls, and 230 of them were upregulated in the patients. Pearson’s correlation scatter plots were used to compare the serum piRNA profiles in patients with brucellosis relative to healthy controls, and the square of the Pearson’s correlation coefficient (R^2^) value for the two groups was 0.713 (P < 0.0001) (**Supplementary Figure 2**). A piRNA was regarded as markedly altered if Illumina SBS technology detected more than 100 reads in either the patients or the controls and if the piRNA exhibited more than a 4-fold difference in expression level between the two groups. According to the above criteria, a total of 86 known piRNAs were significantly differentially expressed between the two groups, with seven upregulated (**Table 1**) and 79 downregulated piRNAs in the patient group (**Supplementary Table 1**).

**Table 1:** Markedly upregulated serum piRNAs in brucellosis patients compared to normal controls determined by Illumina SBS technology.

piRNA	Accession	Count(Control)	Count (Brucellosis)	Fold change (Brucellosis/Control)	*P*-Value
piR-007424	DQ580112	4	113	20.66	2.48×10^-22^
piR-001312	DQ571813	145	3437	17.45	0
piR-002485	DQ573352	15	128	6.28	6.3×10^-17^
piR-016677	DQ592953	27	194	5.30	1.57×10^-22^
piR-016742	DQ593049	29	197	5.00	5.06×10^-22^
piR-000753	DQ570940	47	315	4.94	5.06×10^-34^
piR-020814	DQ598650	19	104	4.02	1.72×10^-10^

### Validity of qRT-PCR measurement system

Subsequently, we conducted qRT-PCR assay to verify the initial screening results obtained using Illumina SBS technology. To ensure the reliability of the qRT-PCR measurement system for piRNAs, we evaluated the quantification accuracy and repeatability of serum RNA isolation and qRT-PCR assays. For the repeatability assay of serum RNA extraction, the Cq values of replicate assays were very similar between the two portions examined (R^2^ = 0.997), indicating that the RNA extraction method was reproducible (**Supplementary Figure 3a**). The analytical reproducibility of the qRT-PCR assay was also excellent (R^2^ = 0.995) (**Supplementary Figure 3b**). Furthermore, 20 replicates of each candidate piRNA were performed to investigate the analytical repeatability of the qRT-PCR assay for candidate piRNAs. The mean CVs for the qRT-PCR assays for piRNAs were 0.39%, 1.17%, and 1.02%, respectively. These results indicate that the qRT-PCR assay used in our study for measuring serum piRNA concentrations was reliable and reproducible.

### Confirmation of Illumina SBS technology results by qRT-PCR assay

We measured the concentrations of seven markedly elevated piRNAs (piR-007424, piR-001312, piR-002485, piR-016677, piR-016742, piR-000753, and piR-020814) that were initially screened in the Illumina SBS technology with a qRT-PCR assay in the training set first. The sequences, general information in different databases, genomic positions of these piRNAs, and their primer and probe sequences are shown in **Supplementary Table 2**, **Supplementary Table 3**, and **Supplementary Table 4**. Among them, the concentrations of three piRNAs, piR-000753, piR-001312, and piR-016742, were found to be higher in the patient group than in the control group (P = 0.0001, P < 0.0001, and P < 0.01, respectively), whereas the concentrations of piR-007424 and piR-016677 were unchanged (**Table 2**). PiR-002485 and piR-020814 were undetectable by qRT-PCR. The fold-changes of piR-000753, piR-001312, and piR-016742 ranged from 3.1-fold to 4.9-fold (**Table 2**).

**Table 2:** Relative concentrations of candidate piRNAs to MIR2911 in the serum samples from brucellosis and control groups determined by individual qRT-PCR assay in the training set and the validation set ^a^.

piRNA	Control	Brucellosis	Fold change (Brucellosis/Control)	*P*-Value ^b^
**Training set**				
Samples, n	27	29		
piR-000753	71.81 ± 13.18	283.77 ± 40.09	3.95	0.0001
piR-001312	2.56 ± 0.41	12.42 ± 2.05	4.86	< 0.0001
piR-016742	2.45 ± 0.35	7.58 ± 1.29	3.09	0.0018
piR-007424	493.27 ± 75.52	845.46 ± 183.65	1.20	0.2250
piR-016677	16.89 ± 3.59	20.35 ± 3.07	1.71	0.7804
**Validation set**				
Samples, n	38	44		
piR-000753	59.88 ± 5.45	190.00 ± 26.84	3.17	< 0.0001
piR-001312	3.00 ± 0.35	10.66 ± 2.27	3.56	0.0010
piR-016742	2.36 ± 0.25	6.69 ± 1.76	2.83	0.0048

a piRNA data are presented as the mean ± SEM (×10^-2^).

b Brucellosis vs. Control.

Subsequently, piR-000753, piR-001312, and piR-016742, were further verified by qRT-PCR in an additional larger cohort (validation set), including 44 brucellosis patients and 38 normal controls. Consistent with the results from the training set, the expression levels of the three piRNAs were also significantly increased in patients in the validation cohort (P < 0.0001, P = 0.001, and P < 0.01, respectively) (**Table 2**). Changes in the concentrations of these piRNAs were greater than 2.8-fold (**Table 2**). Through the above multiphase screening and confirmation, the levels of piR-000753, piR-001312, and piR-016742 in serum from brucellosis patients were confirmed to be steadily expressed and significantly increased compared with normal controls (**Supplementary Figure 4**). Moreover, the serum levels of the three piRNAs also showed no marked differences among patients with different sex, age, stage of the disease, or use of antibiotics (**Supplementary Figure 5**).

### ROC curve and risk score analysis

**Figure 2:**
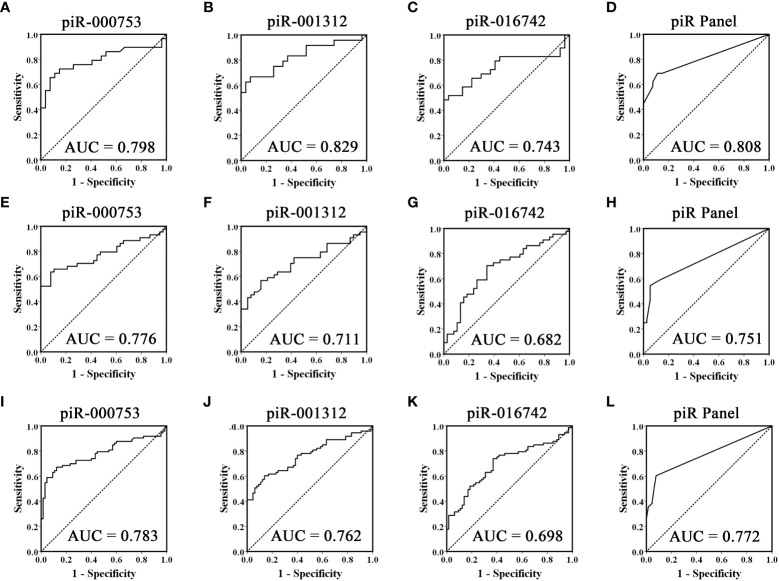
ROC curves analysis of the three selected piRNAs. Receiver operating characteristic (ROC) curves for the ability of three individual piRNAs and the three-piRNA panel to distinguish brucellosis patients from controls in the training set **(A-D)**, validation set **(E-H)** and the combined two sets **(I-L)**.

To evaluate the usefulness of piR-000753, piR-001312, and piR-016742 for distinguishing brucellosis patients from normal controls, we conducted ROC curve analyses and obtained the corresponding AUCs of these three piRNAs ranging from 0.743 to 0.829 in the training set, 0.682 to 0.776 in the validation set, and 0.698 to 0.783 in the combined two sets, respectively (**Figures 2A–C**, **E–G**, and **I–K**). Among the three piRNAs, piR-000753 and piR-001312 exhibited a relatively high diagnostic accuracy (**Figure 2** and **Table 3**).

**Table 3:** Diagnostic efficacy of piR-000753, piR-001312, and piR-016742 to differentiate brucellosis patients from control individuals.

	Training set				Validation set				Total			
Test result piRNA	Sensitivity(%)	Specificity(%)	PPV(%)	NPV(%)	Sensitivity(%)	Specificity(%)	PPV(%)	NPV(%)	Sensitivity(%)	Specificity(%)	PPV(%)	NPV(%)
piR-000753	72.4	85.2	84.0	74.2	70.5	71.1	89.7	66.0	72.6	72.3	87.0	69.0
piR-001312	75.0	74.1	75.0	71.4	63.6	71.1	66.7	60.0	64.4	75.4	70.0	64.7
piR-016742	65.5	77.8	76.0	67.7	70.5	65.8	75.0	55.2	74.0	63.1	75.5	59.5

PPV, positive predictive values; NPV, negative predictive values.

To further assess the diagnostic ability of the three piRNA profiles, we constructed a signature using these three piRNAs by risk score analysis. First, we performed a univariate logistic regression analysis. Based on the risk score analysis, brucellosis status was defined as the dependent variable and the risk score was defined as the concomitant variable. The regression coefficients of these three piRNAs ranged from 1.093 to 3.061, and the odds ratios were larger than 1, ranging from 6.710 to 21.350 (**Supplementary Table 5**), suggesting that these piRNAs are potential risk factors for brucellosis. In addition, we analyzed the diagnostic value of the 3-piRNA panel. We calculated RSF for brucellosis samples and control samples and ranked these samples according to their RSF to divide them into a high-risk group, representing the predicted brucellosis cases, and a low-risk group, representing the predicted control individuals. The frequency tables and ROC curves were then applied to assess the diagnostic value of the three-piRNA panel and to determine the optimal cut-off value. Consequently, the AUCs of this three-piRNA panel were 0.808 (95% CI, 0.690 - 0.925) in the training set (**Figure 2D**) and 0.751(95% CI, 0.645 - 0.858) in the validation set (**Figure 2H**), respectively. When the two sets were calculated together, the AUC was 0.772 (95% CI: 0.693 - 0.851) (**Figure 2L**). At the optimum cut-off value of 2.735, with the value of sensitivity + specificity considered to be maximal, the PPV and NPV of the three-piRNA panel in all samples were 90% and 67%, respectively (**Supplementary Table 6**). These results suggest that the three-piRNA panel has relatively high specificity and PPV for brucellosis detection. Moreover, we also analyzed the diagnostic efficacy of the combinations of random two-piRNAs to differentiate brucellosis patients from control individuals (**Supplementary Table 7**); however, it did not turn out as did the three-piRNA panel (**Supplementary Table 6**).

## Discussion

Human brucellosis is a multi-systemic disease with numerous clinical symptoms but lacks specific manifestations, which still hinders clinicians owing to the limitations of detection methods (Andriopoulos et al., 2007; Kose et al., 2014). Currently, PCR technology is gradually being used for the molecular diagnosis of brucellosis. It has been shown that qRT-PCR techniques are more specific and sensitive than serological tests and could serve as auxiliary tools for brucellosis diagnosis (Sohrabi et al., 2014; Hasanjani Roushan et al., 2016; Sanjuan-Jimenez et al., 2017). piRNAs have been demonstrated to exhibit distinctive profiles in patients with cancers or other diseases, such as lung, pancreatic, and colorectal cancers, male infertility, and Alzheimer’s disease, and are regarded as promising biomarkers for these diseases (Muller et al., 2015; Hong et al., 2016; Reeves et al., 2017; Jun et al., 2017; Weng et al., 2018).

In this study, we performed genome-wide serum piRNA analysis using Illumina SBS technology and observed that the serum piRNA signature of patients with brucellosis was distinct from that of normal controls. For feasibility of clinical testing, we focused on the upregulated piRNAs in the patients in this study. We subsequently conducted qRT-PCR assays to examine the seven upregulated piRNAs arranged in two independent sets and found that the concentrations of piR-000753, piR-001312, and piR-016742 were markedly increased in the sera of brucellosis patients as compared with that of normal controls. Of the three piRNAs, piR-000753 and piR-001312 had larger AUCs than piR-016742, but piR-000753 exhibited a more consistent AUC in both the training and validation sets (**Figure 2**). The PPV and NPV of piR-000753 for the diagnosis of brucellosis were also larger than those of the other two piRNAs (**Table 3**). Moreover, piR-000753 was much more enriched in the serum samples than the other two piRNAs, as shown by their concentrations relative to MIR2911 (**Table 2**). Therefore, piR-000753 may be considered as a potential auxiliary indicator for brucellosis detection. Additionally, we analyzed the diagnostic power of any two piRNA combinations for predicting brucellosis. The three-piRNA panel exhibited a larger AUC than any combination of two piRNAs as well as single piRNAs, including piR-001312 and piR-016742, as shown in **Table 3**, **Supplementary Table 5**, and **Supplementary Table 6**, respectively, suggesting that this three-piRNA panel had the potential to discriminate brucellosis patients from control individuals. We also noticed that both the three-piRNA panel and piR-000753 alone had relatively high PPV, but low NPV for brucellosis detection. We suspect that if these piRNAs were detected in combination with other clinical parameters, it would be possible to improve the diagnostic accuracy of brucellosis. Further studies are necessary to verify this hypothesis.

The normalization of circulating piRNA levels by qRT-PCR is a crucial issue from the beginning. To date, the lack of an established endogenous reference gene or a stable internal control miRNA/piRNA is a major technical issue, which is not negligible in piRNA quantification. To normalize serum piRNAs, we first attempted to use U6 snRNA and miR-16 as endogenous control genes for qRT-PCR analysis; however, our study indicated that there were statistically significant differences in the expression levels of U6 and miR-16 between the two groups (P = 0.0432; P = 0.003) (**Supplementary Figure 6**). Therefore, U6 and miR-16 may not be suitable reference genes for normalizing qRT-PCR expression data of piRNAs. In contrast to tissues or cells, U6 snRNA is easily degraded in serum samples (Peltier and Latham, 2008; Zhang et al., 2010). Although internal miR-16 has been used to normalize serum piRNAs (Lawrie et al., 2008), our previous and present study showed that the expression level of miR-16 itself was altered in certain diseases and cannot be used as a reference control for standardizing serum piRNAs (Chen et al., 2008; Zhang et al., 2010). Based on our previous research methods (Yan et al., 2015; Lu et al., 2017), we used synthetic plant miRNA (MIR2911), an exogenous reference gene, to control for variability in the RNA extraction and purification procedures. MIR2911 could normalize serum piRNAs, as it has no mammalian homologue, and its expression in serum has high repeatability and reproducibility (**Supplementary** **Figure 1**).

The exact reasons for the change in the circulating piRNA expression profile in patients with brucellosis and whether it is involved in the pathological process of brucellosis remain unclear. There are two subtypes of piRNA. One subtype is abundant in germ cells and appears to restrict transposable elements. The major function of this subtype of piRNA is to protect germline and gonadal somatic cells against harmful expression of transposable elements and stabilize the formation of male germ cells (Lee et al., 2011). Brucellosis mainly affects the reproductive system, resulting in miscarriage and infertility in natural host (Atluri et al., 2011). Therefore, some piRNAs may be dysregulated in the reproductive tract and are involved in reproductive diseases caused by brucellosis, thus affecting the expression levels of piRNAs in the circulation. Another subtype of piRNAs originates from genomic regions in somatic cells, such as the brain, liver, and heart, and regulates its target mRNAs, breaking the barrier of restricted expression in germ cells (Lee et al., 2011; Rajan et al., 2014; Qiu et al., 2017). Brucellosis is a systemic disease that affects many tissue types and organs and causes numerous symptoms, such as fever, chills, headache, pain, fatigue, dementia, and arthritis (DelVecchio et al., 2002). Therefore, the change in piRNA expression levels in circulation may be a systemic response to pathological changes in the somatic cells of patients with brucellosis. All pathological changes affected the expression levels of circulating piRNAs throughout the body in both men and women. Further studies are required to clarify this issue.

Although several studies have demonstrated that circulating piRNAs may have great potential as novel non-invasive biomarkers for various diseases, there are still some controversies surrounding circulating piRNAs. One notable issue among these disputes is that piRNAs in circulation may not be “gold standard” piRNAs, but contaminations of longer RNAs or other non-coding RNAs debris. Tosar et al. reported that a subset of piRNAs in a database is possibly independent of the PIWI pathway and may be fragments of non-coding RNA (Tosar et al., 2018). Furthermore, they also demonstrated that one serum piR-54265, which can be used to discriminate colorectal cancer patients from healthy controls, is a small nucleolar RNA (Tosar et al., 2021). These unexpected findings raise the possibility that the three altered piRNAs in brucellosis in this study may also not be canonical piRNA but other small non-coding RNAs that were incorrectly classified as piRNAs. These need to be further verified by bioinformatics tools or experimental verification. Moreover, these lines of new evidence may also counsel us to pay more attention to future research on circulating piRNAs, and targeting piRNAs cannot be identified by simply mapping small RNA-seq data to piRNA databases (Geles et al., 2021).

This study has some limitations. First, the serum piRNA expression profile was only screened and individually confirmed in brucellosis patients and healthy controls, but not in other infection or inflammatory subjects. This may raise the question of whether the three piRNAs were brucellosis-specific or induced by complications of other diseases. Thus, the expression pattern of the three piRNAs should be examined in other bacterial infections or inflammatory subjects to verify their specificity for brucellosis in the future. Second, the use of pooled serum samples for initial piRNA screening might yield inaccurate information due to individual differences; therefore, it will introduce some false-positive results and result in inaccurate information. This can be explained by the fact that only three of the seven piRNAs initially selected for validation showed consistent alterations with the RNA-seq results. Therefore, RNA-seq results must be individually validated using qRT-PCR. Moreover, genome-wide high-throughput screening of individual samples should be encouraged and recommended in similar studies.

In summary, we discovered that the circulating piRNA expression profile in brucellosis serum samples was different from that in normal controls, and three piRNAs (piR-000753, piR-001312, and piR-016742) were highly upregulated in brucellosis patients. Although more studies are required, we demonstrated for the first time that the three-piRNA panel is a promising molecular indicator for brucellosis.

## Data availability statement

The original contributions presented in the study are included in the article/**Supplementary Materials**. Further inquiries can be directed to the corresponding authors.

## Ethics statement

The studies involving human participants were reviewed and approved by ethics committee of Jinling Hospital and Affiliated Hospital of Inner Mongolia Medical University. Informed consent was obtained from all participants. The patients/participants provided their written informed consent to participate in this study.

## Author contributions

Conceptualization: CW, CZ, CYZ, and JJW; data curation: CPZ, CW, QF, NZ, ZZ, and MD; formal analysis: CPZ, ZZ, XC, and FZ; funding acquisition: CW and CZ; methodology: CW, CPZ, ZZ, and CZ; resources: JJW, CYZ, QF, and FZ; supervision: CZ, CYZ, and JJW; roles/writing: original draft: CPZ, CW, and CZ; writing and review and editing: JJW and CYZ. All authors contributed to the article and approved the submitted version.

## Funding

This work was supported by grants from National Natural Science Foundation of China (no. 81772282 and 82072376), Fund of State Key Laboratory of Analytical Chemistry for Life Science (no. 5431ZZXM1907), Natural Science Foundation of Jiangsu Provincial (BK20211132), and Fund of Jiangsu Postdoctoral Science Foundation (2021K322C). The funder played no role in the study.

## Conflict of interest

The authors declare that the research was conducted in the absence of any commercial or financial relationships that could be construed as a potential conflict of interest.

## Publisher’s note

All claims expressed in this article are solely those of the authors and do not necessarily represent those of their affiliated organizations, or those of the publisher, the editors and the reviewers. Any product that may be evaluated in this article, or claim that may be made by its manufacturer, is not guaranteed or endorsed by the publisher.

## Abbreviations

piRNA, Piwi-interacting RNA; qRT-PCR, quantitative reverse transcription polymerase chain reaction; Illumina SBS technology, the sequencing by synthesis (SBS) technology of Illumina; Cq, quantification cycle; AUC, the area under the ROC curve; PPV, positive predictive value; NPV, negative predictive value; ELISA, enzyme linked immunosorbent assay.
