# Supplementary material for: Increased serum piwi-interacting RNAs as a novel potential diagnostic tool for brucellosis
Source: Front Cell Infect Microbiol. 2022 Sep 15;12:992775. doi: 10.3389/fcimb.2022.992775 (PMC9519857; doi:10.3389/fcimb.2022.992775)
Supplement: Supplementary file 1 [file DataSheet_1.docx]

**Increased serum piwi-interacting RNAs as a novel potential diagnostic tool for brucellosis**

Cheng Wang^1,2,^^†^ , Cuiping Zhang^1,2,^^3,†^, Quan Fu^4,5,†^, Nan Zhang^1^, Meng Ding^1,2^, Zhen Zhou^2^, Xi Chen^2^, Fengmin Zhang^4^, Chunni Zhang^1,2,*^, Chen-Yu Zhang^1,2,*^ & Jun-Jun Wang^1,2,*^

^1^ Department of Clinical Laboratory, Jinling Hospital, State Key Laboratory of Analytical Chemistry for Life Science, NJU Advanced Institute for Life Sciences (NAILS), School of Life Sciences, Nanjing University, Nanjing, 210002, China;

^2^ Nanjing Drum Tower Hospital Center of Molecular Diagnostic and Therapy, Chinese Academy of Medical Sciences Research Unit of Extracellular RNA, State Key Laboratory of Pharmaceutical Biotechnology, Jiangsu Engineering Research Center for MicroRNA Biology and Biotechnology, NJU Advanced Institute of Life Sciences (NAILS), Institute of Artificial Intelligence Biomedicine, School of Life Sciences, Nanjing University, Nanjing, 210023, China;

^3^ Department of Prenatal Diagnosis, Women's Hospital of Nanjing Medical University, Nanjing Maternity and Child Health care Hospital, Nanjing, China

^4^ Department of Microbiology, Harbin Medical University, Harbin, 150081, China;

^5^ Department of Clinical Laboratory, Affiliated Hospital of Inner Mongolia Medical University, Hohhot, 010050, China.

^*^ Corresponding authors (Jun-Jun Wang, email: [wangjunjun9202@163.com](mailto:wangjunjun9202@163.com); Chunni Zhang, email: [zchunni27@hotmail.com](mailto:zchunni27@hotmail.com); Chen-Yu Zhang, email: [cyzhang@nju.edu.cn](mailto:cyzhang@nju.edu.cn))

^†^ Contributed equally to this work.

**Supplementary Material:**

**1 Supplementary Methods**

**1.1 RNA isolation of serum**

For the Illumina SBS technology, an equal volume of serum from each participant was pooled separately to form case and control sample pools (each pool contained 20 mL serum). Total RNA was extracted from each pooled serum sample using TRIzol reagent (Invitrogen, MA, USA). The aqueous phase was subjected to 3 steps of acid phenol/chloroform purification to eliminate protein residues before isopropyl alcohol precipitation. The resulting RNA pellet was dissolved in 20 μL RNase-free water and stored at -80°C until further analysis.

For the RT-qPCR assay, total RNA was extracted from 100 μL serum with a 1-step phenol/chloroform purification protocol, which was mixed with 300 μL deionized water, 200 μL acid phenol, 20μL exogenous MIR2911, and 200 μL chloroform. The mixture was vortex-mixed vigorously and centrifuged at room temperature for 15 min. After phase separation, the aqueous layer was mixed with 1.5 volumes of isopropyl alcohol and 0.1 volumes of 3 mol/L sodium acetate (pH = 5.3). This solution was stored at -20 °C for 1 h at least. The RNA pellet was collected by centrifugation at 16,000g for 20 min at 4 °C. The resulting RNA pellet was washed once with 750 mL/L ethanol and dried for 10 min at room temperature. Finally, the pellet was dissolved in 30 μL of RNase-free water and stored at -80 °C until further analysis.

**1.2 Illumina sequencing via synthesis (SBS) technology of serum piRNAs**

Illumina SBS technology was performed as follows. Briefly, after PAGE purification of small RNA molecules (< 50 nucleotides) and ligation of a pair of adaptors to the RNA 5′ and 3′ ends, RNA molecules were amplified using primers to the adaptor regions for 17 cycles. Fragments that were approximately 90 bp (small RNA + adaptors) were isolated from an agarose gel. Purified DNA was used for cluster generation and sequencing analysis using the Illumina’s Sequencer according to the manufacturer’s instructions. Image files were generated by the sequencer and were processed to produce digital-quality data by CASAVA. Low quality sequences, 5′ adapter and polyA contamination were removed. After masking adapter sequences, clean reads were used for further analysis. Unique sequences with lengths of 17 - 44 nucleotides were firstly mapped to Brucella genome and the unmapped sequences were then aligned to human genomes. The mapped reads were used for further analysis. These sequences were then annotated by using miRBase, tRNA, rRNA and rfam database, respectively. The unmapped reads were finally mapped to piRNABank to get the piRNA expression profile.

**1.3 Assay precision of RT-qPCR for piRNA**

For the repeatability of RNA extraction, pooled serum from 10 healthy individuals was divided into two identical parts (each 100 μL), and RNA was extracted from the two pooled serum samples, respectively. Then the amounts of five piRNAs (including piR-007424, piR-001312, piR-016677, piR-016742 and piR-000753) ranging from low to high levels were measured with RT-qPCR assay. Each sample was measured in triplicate.

The analytical repeatability of the RT-qPCR assay, exclusive of RNA extraction, was evaluated by detecting the same five piRNAs as above in the two same RNA preparations extracted from pooled serum collected from 10 healthy individuals. Each sample was conducted in triplicate.

The assay precision of the piRNAs quantification, inclusive of RNA extraction, was evaluated by respectively performing quantification of 20 replicates of piR-000753, piR-001312 and piR-016742.

**2 Supplementary Tables and Figures**

**2.1 Supplementary Tables**

**Supplementary Table 1.** Down-regulated piRNAs in serum samples from brucellosis patients compared to normal controls determined by Illumina SBS technology.

| piRNA | Accession | Reads Count  (Control) | Reads Count  (Brucellosis) | Fold change  (Brucellosis/Control) |
| --- | --- | --- | --- | --- |
| piR-020575 | [DQ598300](http://www.ncbi.nlm.nih.gov/entrez/viewer.fcgi?db=nucleotide&val=DQ598300) | 1333 | 6 | -299.781 |
| piR-020541 | [DQ598252](http://www.ncbi.nlm.nih.gov/entrez/viewer.fcgi?db=nucleotide&val=DQ598252) | 1571 | 28 | -76.392 |
| piR-004080 | [DQ575563](http://www.ncbi.nlm.nih.gov/entrez/viewer.fcgi?db=nucleotide&val=DQ575563) | 316 | 7 | -61.459 |
| piR-000172 | [DQ570169](http://www.ncbi.nlm.nih.gov/entrez/viewer.fcgi?db=nucleotide&val=DQ570169) | 305 | 7 | -59.324 |
| piR-023085 | [DQ601565](http://www.ncbi.nlm.nih.gov/entrez/viewer.fcgi?db=nucleotide&val=DQ601565) | 107 | 3 | -48.125 |
| piR-001042 | [DQ571335](http://www.ncbi.nlm.nih.gov/entrez/viewer.fcgi?db=nucleotide&val=DQ571335) | 235 | 7 | -45.703 |
| piR-020009 | [DQ597484](http://www.ncbi.nlm.nih.gov/entrez/viewer.fcgi?db=nucleotide&val=DQ597484) | 899 | 29 | -42.013 |
| piR-015151 | [DQ590705](http://www.ncbi.nlm.nih.gov/entrez/viewer.fcgi?db=nucleotide&val=DQ590705) | 326 | 12 | -36.656 |
| piR-019912 | [DQ597341](http://www.ncbi.nlm.nih.gov/entrez/viewer.fcgi?db=nucleotide&val=DQ597341) | 7824 | 336 | -31.617 |
| piR-003832 | [DQ575213](http://www.ncbi.nlm.nih.gov/entrez/viewer.fcgi?db=nucleotide&val=DQ575213) | 1600 | 75 | -28.932 |
| piR-003678 | [DQ574997](http://www.ncbi.nlm.nih.gov/entrez/viewer.fcgi?db=nucleotide&val=DQ574997) | 207 | 10 | -28.113 |
| piR-001184 | [DQ574997](http://www.ncbi.nlm.nih.gov/entrez/viewer.fcgi?db=nucleotide&val=DQ574997) | 927 | 47 | -26.795 |
| piR-004308 | [DQ575882](http://www.ncbi.nlm.nih.gov/entrez/viewer.fcgi?db=nucleotide&val=DQ575882) | 100 | 5 | -26.667 |
| piR-020492 | [DQ598170](http://www.ncbi.nlm.nih.gov/entrez/viewer.fcgi?db=nucleotide&val=DQ598170) | 21427 | 1101 | -26.424 |
| piR-017355 | [DQ593995](http://www.ncbi.nlm.nih.gov/entrez/viewer.fcgi?db=nucleotide&val=DQ593995) | 246 | 13 | -25.652 |
| piR-000651 | [DQ570812](http://www.ncbi.nlm.nih.gov/entrez/viewer.fcgi?db=nucleotide&val=DQ570812) | 149 | 9 | -22.333 |
| piR-022421 | [DQ600670](http://www.ncbi.nlm.nih.gov/entrez/viewer.fcgi?db=nucleotide&val=DQ600670) | 22873 | 1611 | -19.278 |
| piR-008982 | [DQ582174](http://www.ncbi.nlm.nih.gov/entrez/viewer.fcgi?db=nucleotide&val=DQ582174) | 473 | 40 | -16.057 |
| piR-004153 | [DQ575660](http://www.ncbi.nlm.nih.gov/entrez/viewer.fcgi?db=nucleotide&val=DQ575660) | 5352 | 463 | -15.696 |
| piR-022315 | [DQ600539](http://www.ncbi.nlm.nih.gov/entrez/viewer.fcgi?db=nucleotide&val=DQ600539) | 1354 | 118 | -15.592 |
| piR-010862 | [DQ584649](http://www.ncbi.nlm.nih.gov/entrez/viewer.fcgi?db=nucleotide&val=DQ584649) | 2422 | 219 | -15.014 |
| piR-008927 | [DQ582108](http://www.ncbi.nlm.nih.gov/entrez/viewer.fcgi?db=nucleotide&val=DQ582108) | 163 | 15 | -14.662 |
| piR-022294 | [DQ600513](http://www.ncbi.nlm.nih.gov/entrez/viewer.fcgi?db=nucleotide&val=DQ600513) | 224 | 21 | -14.523 |
| piR-008983 | [DQ582175](http://www.ncbi.nlm.nih.gov/entrez/viewer.fcgi?db=nucleotide&val=DQ582175) | 7677 | 739 | -14.105 |
| piR-009981 | [DQ583434](http://www.ncbi.nlm.nih.gov/entrez/viewer.fcgi?db=nucleotide&val=DQ583434) | 4370 | 426 | -13.929 |
| piR-019723 | [DQ597069](http://www.ncbi.nlm.nih.gov/entrez/viewer.fcgi?db=nucleotide&val=DQ597069) | 284 | 28 | -13.811 |
| piR-008026 | [DQ580917](http://www.ncbi.nlm.nih.gov/entrez/viewer.fcgi?db=nucleotide&val=DQ580917) | 531 | 56 | -12.869 |
| piR-020453 | [DQ598108](http://www.ncbi.nlm.nih.gov/entrez/viewer.fcgi?db=nucleotide&val=DQ598108) | 1687 | 194 | -11.810 |
| piR-022437 | [DQ600690](http://www.ncbi.nlm.nih.gov/entrez/viewer.fcgi?db=nucleotide&val=DQ600690) | 24797 | 2878 | -11.699 |
| piR-003817 | [DQ575190](http://www.ncbi.nlm.nih.gov/entrez/viewer.fcgi?db=nucleotide&val=DQ575190) | 337 | 41 | -11.175 |
| piR-022017 | [DQ600174](http://www.ncbi.nlm.nih.gov/entrez/viewer.fcgi?db=nucleotide&val=DQ600174) | 2604 | 322 | -10.979 |
| piR-014954 | [DQ590447](http://www.ncbi.nlm.nih.gov/entrez/viewer.fcgi?db=nucleotide&val=DQ590447) | 119 | 15 | -10.700 |
| piR-022296 | [DQ600515](http://www.ncbi.nlm.nih.gov/entrez/viewer.fcgi?db=nucleotide&val=DQ600515) | 46955 | 5995 | -10.634 |
| piR-023338 | [DQ601914](http://www.ncbi.nlm.nih.gov/entrez/viewer.fcgi?db=nucleotide&val=DQ601914) | 2039 | 263 | -10.527 |
| piR-005799 | [DQ577967](http://www.ncbi.nlm.nih.gov/entrez/viewer.fcgi?db=nucleotide&val=DQ577967) | 6795 | 879 | -10.496 |
| piR-016374 | DQ592313 | 961 | 130 | -10.038 |
| piR-017008 | [DQ593502](http://www.ncbi.nlm.nih.gov/entrez/viewer.fcgi?db=nucleotide&val=DQ593502) | 1075 | 153 | -9.540 |
| piR-008488 | [DQ581533](http://www.ncbi.nlm.nih.gov/entrez/viewer.fcgi?db=nucleotide&val=DQ581533) | 837 | 121 | -9.398 |
| piR-019752 | [DQ597110](http://www.ncbi.nlm.nih.gov/entrez/viewer.fcgi?db=nucleotide&val=DQ597110) | 8915 | 1303 | -9.289 |
| piR-014880 | [DQ590349](http://www.ncbi.nlm.nih.gov/entrez/viewer.fcgi?db=nucleotide&val=DQ590349) | 294 | 43 | -9.281 |
| piR-010024 | [DQ583491](http://www.ncbi.nlm.nih.gov/entrez/viewer.fcgi?db=nucleotide&val=DQ583491) | 2814 | 414 | -9.231 |
| piR-015428 | [DQ591080](http://www.ncbi.nlm.nih.gov/entrez/viewer.fcgi?db=nucleotide&val=DQ591080) | 243 | 36 | -9.157 |
| piR-022295 | DQ600514 | 2597 | 402 | -8.771 |
| piR-018105 | [DQ594940](http://www.ncbi.nlm.nih.gov/entrez/viewer.fcgi?db=nucleotide&val=DQ594940) | 399 | 63 | -8.599 |
| piR-015149 | [DQ590703](http://www.ncbi.nlm.nih.gov/entrez/viewer.fcgi?db=nucleotide&val=DQ590703) | 268 | 43 | -8.461 |
| piR-018800 | [DQ595833](http://www.ncbi.nlm.nih.gov/entrez/viewer.fcgi?db=nucleotide&val=DQ595833) | 261 | 42 | -8.422 |
| piR-008921 | [DQ582102](http://www.ncbi.nlm.nih.gov/entrez/viewer.fcgi?db=nucleotide&val=DQ582102) | 159 | 26 | -8.290 |
| piR-005023 | [DQ576923](http://www.ncbi.nlm.nih.gov/entrez/viewer.fcgi?db=nucleotide&val=DQ576923) | 631 | 105 | -8.153 |
| piR-014633 | [DQ590027](http://www.ncbi.nlm.nih.gov/entrez/viewer.fcgi?db=nucleotide&val=DQ590027) | 277 | 47 | -8.008 |
| piR-013745 | [DQ588779](http://www.ncbi.nlm.nih.gov/entrez/viewer.fcgi?db=nucleotide&val=DQ588779) | 21566 | 3760 | -7.788 |
| piR-020786 | [DQ598612](http://www.ncbi.nlm.nih.gov/entrez/viewer.fcgi?db=nucleotide&val=DQ598612) | 1411 | 246 | -7.788 |
| piR-011374 | [DQ585363](http://www.ncbi.nlm.nih.gov/entrez/viewer.fcgi?db=nucleotide&val=DQ585363) | 167 | 31 | -7.329 |
| piR-004262 | [DQ575814](http://www.ncbi.nlm.nih.gov/entrez/viewer.fcgi?db=nucleotide&val=DQ575814) | 1089 | 202 | -7.317 |
| piR-013393 | [DQ588158](http://www.ncbi.nlm.nih.gov/entrez/viewer.fcgi?db=nucleotide&val=DQ588158) | 318 | 59 | -7.313 |
| piR-000848 | [DQ571067](http://www.ncbi.nlm.nih.gov/entrez/viewer.fcgi?db=nucleotide&val=DQ571067) | 671 | 125 | -7.284 |
| piR-009154 | [DQ582400](http://www.ncbi.nlm.nih.gov/entrez/viewer.fcgi?db=nucleotide&val=DQ582400) | 3548 | 671 | -7.179 |
| piR-015800 | [DQ591595](http://www.ncbi.nlm.nih.gov/entrez/viewer.fcgi?db=nucleotide&val=DQ591595) | 4398 | 845 | -7.067 |
| piR-011187 | [DQ585094](http://www.ncbi.nlm.nih.gov/entrez/viewer.fcgi?db=nucleotide&val=DQ585094) | 246 | 49 | -6.808 |
| piR-003384 | [DQ574582](http://www.ncbi.nlm.nih.gov/entrez/viewer.fcgi?db=nucleotide&val=DQ574582) | 116 | 24 | -6.575 |
| piR-014959 | [DQ590455](http://www.ncbi.nlm.nih.gov/entrez/viewer.fcgi?db=nucleotide&val=DQ590455) | 1960 | 411 | -6.474 |
| piR-022298 | [DQ600517](http://www.ncbi.nlm.nih.gov/entrez/viewer.fcgi?db=nucleotide&val=DQ600517) | 6036 | 1299 | -6.310 |
| piR-003782 | [DQ575134](http://www.ncbi.nlm.nih.gov/entrez/viewer.fcgi?db=nucleotide&val=DQ575134) | 146 | 32 | -6.182 |
| piR-015150 | [DQ590704](http://www.ncbi.nlm.nih.gov/entrez/viewer.fcgi?db=nucleotide&val=DQ590704) | 1095 | 260 | -5.719 |
| piR-002441 | [DQ573293](http://www.ncbi.nlm.nih.gov/entrez/viewer.fcgi?db=nucleotide&val=DQ573293) | 1243 | 302 | -5.588 |
| piR-005337 | [DQ577347](http://www.ncbi.nlm.nih.gov/entrez/viewer.fcgi?db=nucleotide&val=DQ577347) | 6372 | 1622 | -5.334 |
| piR-018735 | [DQ595749](http://www.ncbi.nlm.nih.gov/entrez/viewer.fcgi?db=nucleotide&val=DQ595749) | 1020 | 263 | -5.266 |
| piR-017138 | [DQ593692](http://www.ncbi.nlm.nih.gov/entrez/viewer.fcgi?db=nucleotide&val=DQ593692) | 377 | 100 | -5.119 |
| piR-020401 | [DQ598029](http://www.ncbi.nlm.nih.gov/entrez/viewer.fcgi?db=nucleotide&val=DQ598029) | 1244 | 335 | -5.041 |
| piR-008981 | [DQ582173](http://www.ncbi.nlm.nih.gov/entrez/viewer.fcgi?db=nucleotide&val=DQ582173) | 136 | 37 | -4.995 |
| piR-013359 | [DQ588112](http://www.ncbi.nlm.nih.gov/entrez/viewer.fcgi?db=nucleotide&val=DQ588112) | 120 | 34 | -4.800 |
| piR-014626 | [DQ590020](http://www.ncbi.nlm.nih.gov/entrez/viewer.fcgi?db=nucleotide&val=DQ590020) | 270 | 77 | -4.762 |
| piR-014636 | [DQ590030](http://www.ncbi.nlm.nih.gov/entrez/viewer.fcgi?db=nucleotide&val=DQ590030) | 280 | 82 | -4.632 |
| piR-004735 | [DQ576498](http://www.ncbi.nlm.nih.gov/entrez/viewer.fcgi?db=nucleotide&val=DQ576498) | 144 | 43 | -4.544 |
| piR-014635 | [DQ590029](http://www.ncbi.nlm.nih.gov/entrez/viewer.fcgi?db=nucleotide&val=DQ590029) | 292 | 88 | -4.509 |
| piR-009901 | [DQ583332](http://www.ncbi.nlm.nih.gov/entrez/viewer.fcgi?db=nucleotide&val=DQ583332) | 177 | 54 | -4.455 |
| piR-011186 | [DQ585093](http://www.ncbi.nlm.nih.gov/entrez/viewer.fcgi?db=nucleotide&val=DQ585093) | 291 | 90 | -4.390 |
| piR-019485 | [DQ596756](http://www.ncbi.nlm.nih.gov/entrez/viewer.fcgi?db=nucleotide&val=DQ596756) | 118 | 38 | -4.224 |
| piR-010023 | [DQ583490](http://www.ncbi.nlm.nih.gov/entrez/viewer.fcgi?db=nucleotide&val=DQ583490) | 872 | 285 | -4.154 |
| piR-019544 | [DQ596830](http://www.ncbi.nlm.nih.gov/entrez/viewer.fcgi?db=nucleotide&val=DQ596830) | 791 | 267 | -4.023 |

**Supplementary Table 2.** The sequences of markedly up-regulated piRNAs in brucellosis patients by Illumina SBS technology and their general information in different databases.

| piRNABank ID | Accession | Chromosome | Sequence | length |
| --- | --- | --- | --- | --- |
| piR-000753 | DQ570940 | 17 | AGCAGUUGAACAUGGGUCAGUCGGUCCUG | 29 |
| piR-001312 | DQ571813 | 1,2,5,6,16,17 | AUUGGUGGUUCAGUGGUAGAAUUCUCGCCUG | 31 |
| piR-002485 | DQ573352 | 7,X,Y | UCAGACAUUUGGUGUAUGUGCUUGGC | 26 |
| piR-016742 | DQ593049 | 1,5,16 | CCGGCUAGCUCAGUCGGUAGAGCAUGAGA | 29 |
| piR-016677 | DQ592953 | 2 | CCCCUGGUGGUCUAGUGGUUAGGAUUCGGC | 30 |
| piR-007424 | DQ580112 | 8 | UGAGAACUAGCUAAACAGGGUCGGGCAGA | 29 |
| piR-020814 | DQ598650 | 22 | GUUCAGUGAUGAGGCCUGGAAUGUGCGCUGGG | 32 |
| piRBase ID | **Accession** | **Aliases** | **Sequence** | **length** |
| piR-hsa-1191 | DQ570940 | piR-31052 | AGCAGTTGAACATGGGTCAGTCGGTCCTG | 29 |
| piR-hsa-2107 | DQ571813 | piR-31925 | ATTGGTGGTTCAGTGGTAGAATTCTCGCCTG | 31 |
| piR-hsa-3645 | DQ573352 | piR-41464 | TCAGACATTTGGTGTATGTGCTTGGC | 26 |
| piR-hsa-23327 | DQ593049 | piR-33161 | CCGGCTAGCTCAGTCGGTAGAGCATGAGA | 29 |
| piR-hsa-23231 | DQ592953 | piR-33065 | CCCCTGGTGGTCTAGTGGTTAGGATTCGGC | 30 |
| piR-hsa-10352 | DQ580112 | piR-48224 | TGAGAACTAGCTAAACAGGGTCGGGCAGA | 29 |
| piR-hsa-28850 | DQ598650 | piR-36716 | GTTCAGTGATGAGGCCTGGAATGTGCGCTGGG | 32 |
| piRNAQuest ID | **Accession** | **Aliases** | **Sequence** | **length** |
| hsa_piRNA_31019 | DQ570940.1 | gi\|108052194, piR-31052 | AGCAGTTGAACATGGGTCAGTCGGTCCTG | 29 |
| hsa_piRNA_30146 | DQ571813.1 | gi\|108053067, piR-31925 | ATTGGTGGTTCAGTGGTAGAATTCTCGCCTG | 31 |
| hsa_piRNA_20607 | DQ573352.1 | gi\|108054606, piR-41464 | TCAGACATTTGGTGTATGTGCTTGGC | 26 |
| hsa_piRNA_28910 | DQ593049.1 | gi\|108085634, piR-33161 | CCGGCTAGCTCAGTCGGTAGAGCATGAGA | 29 |
| hsa_piRNA_29006 | DQ592953.1 | gi\|108085453, piR-33065 | CCCCTGGTGGTCTAGTGGTTAGGATTCGGC | 30 |
| hsa_piRNA_13847 | DQ580112.1 | gi\|108066469, piR-48224 | TGAGAACTAGCTAAACAGGGTCGGGCAGA | 29 |
| hsa_piRNA_25355 | DQ598650.1 | gi\|108096295, piR-36716 | GTTCAGTGATGAGGCCTGGAATGTGCGCTGGG | 32 |

**Supplementary Table 3.** The chromosomal positions of identified piRNAs.

| **piRNABank ID** | **Accession** | **Chromosome** | **Genomic position** |
| --- | --- | --- | --- |
| piR-000753 | DQ570940 | 17 | 19296281:19296309   (NCBI36) |
| piR-001312 | DQ571813 | 1 | 159760299-159760329 [Minus]    (NCBI36) |
|  |  | 16 | 69369653-69369683 [Minus]    (NCBI36) |
|  |  | 16 | 69370481-69370511 [Minus]    (NCBI36) |
|  |  | 16 | 69380100-69380130 [Plus]    (NCBI36) |
|  |  | 16 | 69380913-69380943 [Plus]    (NCBI36) |
|  |  | 17 | 7969791-7969821 [Plus]    (NCBI36) |
|  |  | 2 | 156965943-156965973 [Minus]    (NCBI36) |
|  |  | 5 | 85704428-85704458 [Minus]    (NCBI36) |
|  |  | 6 | 27978703-27978733 [Minus]    (NCBI36) |
| piR-016742 | DQ593049 | 1 | 144106921-144106949 [Minus]    (NCBI36) |
|  |  | 16 | 3147449-3147477 [Minus]    (NCBI36) |
|  |  | 16 | 3165695-3165723 [Plus]    (NCBI36) |
|  |  | 5 | 180567363-180567391 [Plus]    (NCBI36) |
|  |  | 5 | 180581627-180581655 [Minus]    (NCBI36) |

**Supplementary Table 4.** The primer and probe sequence of identified piRNAs.

| piRNA | Sequence |
| --- | --- |
| piR-000753 | forward primer: CCGTAGCAGTTGAACATGGGT |
|  | reverse primer: TATGGTTGTAGACGACTCCTTGAC |
|  | probe: CCCTATCCAACCATACAGACCAGGACC |
| piR-001312 | forward primer: AATCCATTGGTGGTTCAGTG |
|  | reverse primer: CAGAGAAGAGTCCGAGCATT |
|  | probe: TGCCCTCTGGCTTCGTCTAGCCAGTC |
| piR-016742 | forward primer: CCATTCCGGCTAGCTCAGTC |
|  | reverse primer: TATGCTTGTTCACGAGTCCTTGTC |
|  | probe: CATCCCTATCCAAGCATACAGACTCTC |

**Supplementary Table 5.** The odds ratio of the 3 candidate piRNAs in univariate logistic regression model analysis for brucellosis patients and normal controls.

| piRNAs | B | Std. Error | OR | 95% CI for OR | | P-Value |
| --- | --- | --- | --- | --- | --- | --- |
|  |  |  |  | **Lower** | **Upper** |  |
| piR-000753 | 3.061 | 0.569 | 21.350 | 7.002 | 65.097 | < 0.0001 |
| piR-001312 | 2,558 | 0.568 | 12.904 | 4.241 | 39.263 | < 0.0001 |
| piR-016742 | 1.904 | 0.576 | 6.710 | 2.170 | 20.752 | < 0.0001 |

B, regression coefficient; OR, odds ratio.

**Supplementary Table 6.** Risk score analysis of brucellosis cases and control subjects on the three-piRNA panel.

|  | **Group** | **AUC** | **95% CI** | **Cut-off value**  **(0 ~ 2.735)** | **Cut-off value**  **(2.735~8.487)** | **Sensitivity**  **(%)** | **Specificity**  **(%)** | **PPV**  **(%)** | **NPV**  **(%)** |
| --- | --- | --- | --- | --- | --- | --- | --- | --- | --- |
| Training set | Control | 0.808 | (0.690-0.925) | 24 | 3 | 0.655 | 0.862 |  | 0.73 |
|  | Brucellosis |  |  | 9 | 20 |  |  | 0.87 |  |
| Validation set | Control | 0.751 | (0.645-0.858) | 36 | 2 | 0.591 | 0.868 |  | 0.64 |
|  | Brucellosis |  |  | 20 | 24 |  |  | 0.92 |  |
| Total | Control | 0.772 | (0.693-0.851) | 60 | 5 | 0.630 | 0.862 |  | 0.67 |
|  | Brucellosis |  |  | 29 | 44 |  |  | 0.90 |  |

AUC, area under ROC curve; 95% CI, 95% confidence interval; PPV, positive predictive values; NPV, negative predictive value

**Supplementary Table 7.** Diagnostic efficacy of the combinations of two piRNAs to differentiate brucellosis patients from control individuals.

|  | **piR-000753 + piR-001312** | | | | **piR-000753 + piR-016742** | | | | **piR-001312 + piR-016742** | | | |
| --- | --- | --- | --- | --- | --- | --- | --- | --- | --- | --- | --- | --- |
| Group | AUC | 95% CI | Sensitivity  (%) | Specificity  (%) | AUC | 95% CI | Sensitivity  (%) | Specificity  (%) | AUC | 95% CI | Sensitivity  (%) | Specificity  (%) |
| Training set | 0.806 | (0.687-0.925) | 69.0 | 88.9 | 0.789 | (0.667-0.911) | 65.5 | 85.2 | 0.774 | (0.648-0.900) | 62.1 | 88.9 |
| Validation set | 0.749 | (0.643-0.856) | 54.5 | 94.7 | 0.684 | (0.569-0.799) | 43.2 | 89.5 | 0.685 | (0.570-0.801) | 47.7 | 86.8 |
| Total | 0.770 | (0.691-0.850) | 60.3 | 92.3 | 0.724 | (0.639-0.809) | 52.1 | 87.7 | 0.721 | (0.635-0.806) | 53.4 | 87.7 |

AUC, area under ROC curve; 95% CI, 95% confidence interval.

**2.2 Supplementary Figures**


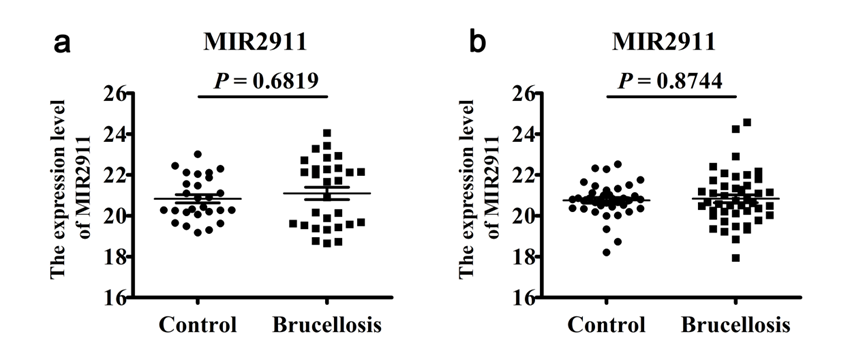


**Supplementary Figure 1.** The expression level of MIR2911. The serum levels of MIR2911 in the training set (a) and the validation set (b) measured by qRT-PCR assay.





**Supplementary Figure 2.** Pearson’s correlation scatter plot of serum piRNA levels in the brucellosis group and control group as determined by Illumina SBS technology, *P* < 0.0001.


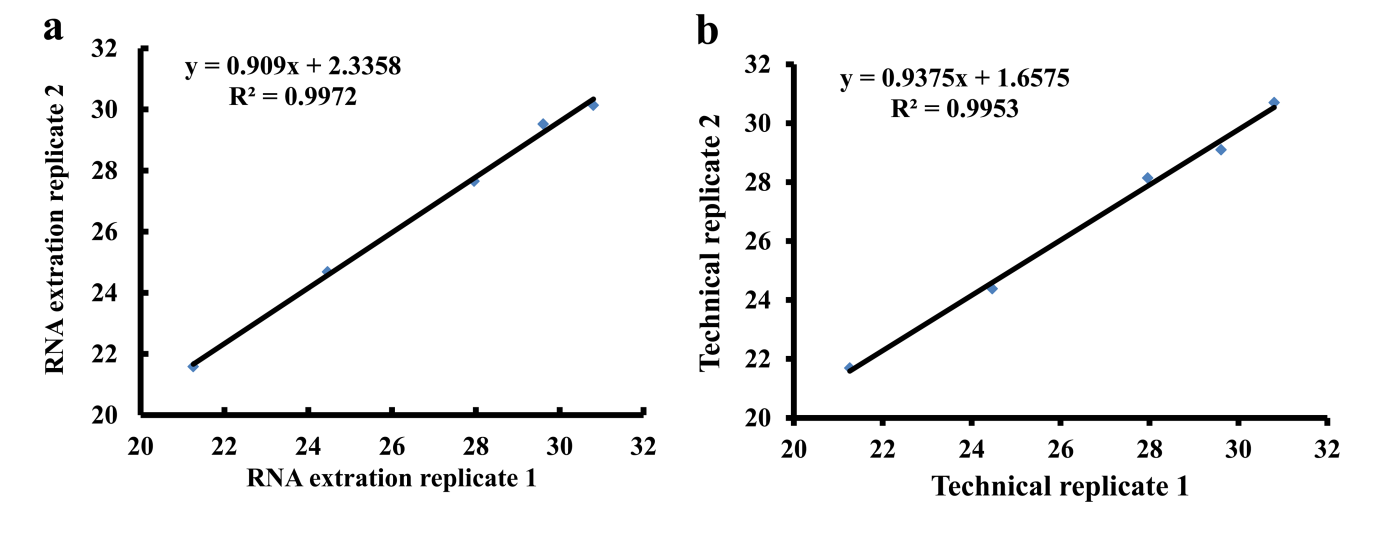


**Supplementary Figure 3.** The repeatability of serum RNA extraction and qRT-PCR assay for piRNAs. The repeatability of serum RNA extraction for the analytical characterization of qRT-PCR assay (a) and the repeatability of qRT-PCR assay for piRNAs (b).


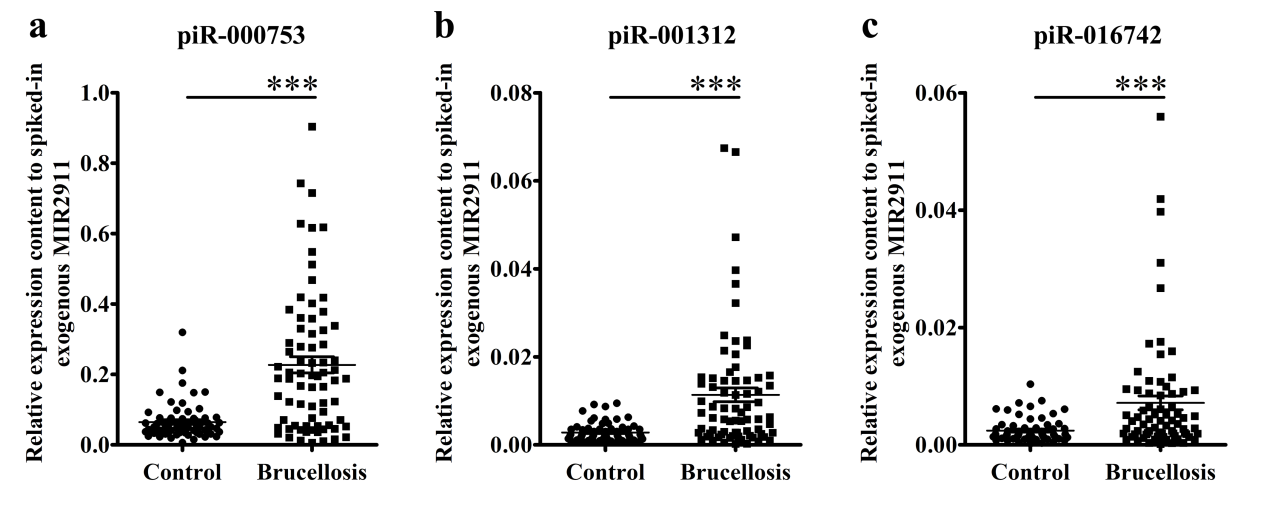


**Supplementary Figure 4.** The relative expression levels of candidate piRNAs in the combined two sets. The serum levels of piR-000753, piR-001312, piR-016742 in all subjects (Control = 65, Brucellosis = 73) (a-c). The relative content of piRNA was calculated using the 2^−∆Cq^ method. Each *P*-value was derived from a nonparametric Mann–Whitney U-test. ****P* < 0.001.


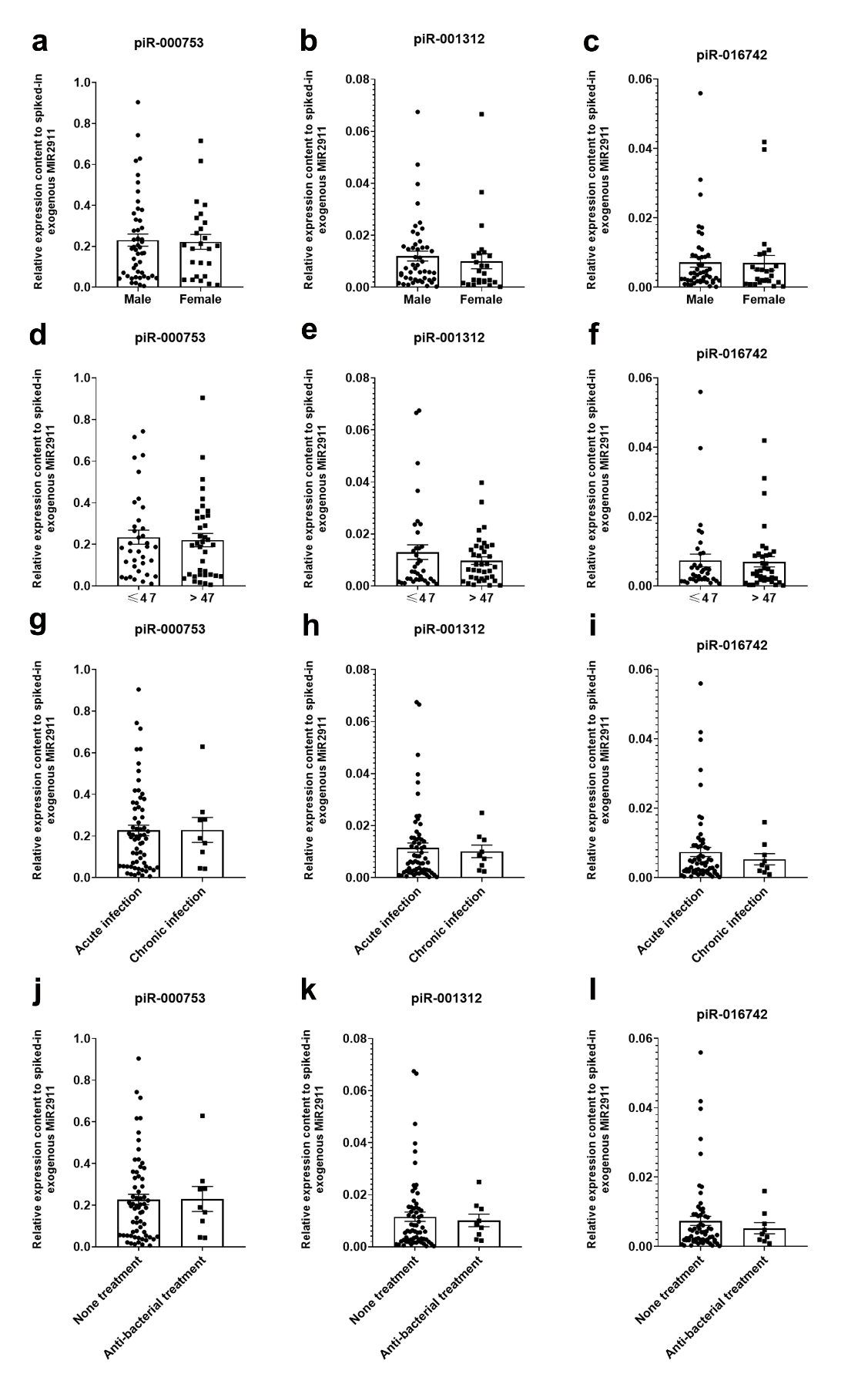


**Supplementary Figure 5.** The relative expression levels of candidate piRNAs among patients’ groups that were divided with gender, age, disease stage, antibiotics use. The serum levels of piR-000753, piR-001312, piR-016742 in male and female patients (male = 48, female = 25) (a-c), in patients with different age (age ≤ 47 years = 36, age > 47 years = 37) (d-f), patients with acute infection (n = 64) or chronic infection (n = 9) (g-i), and patients with (n = 9) or without (n = 64) antibiotics treatment (j-l). The relative content of piRNA was calculated using the 2^−∆Cq^ method.


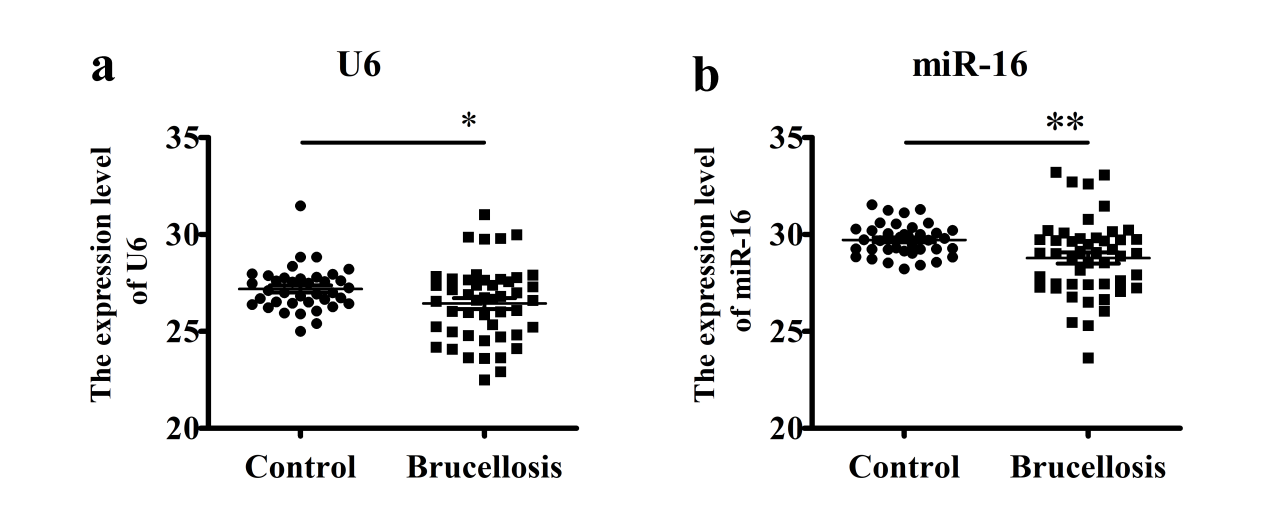


**Supplementary Figure 6.** The expression level of U6 and miR-16. The expression level of U6 (a) and miR-16 (b) in brucellosis patients (n = 48) and normal controls (n = 38) analyzed by qRT-PCR assay. **P* < 0.05, ***P* < 0.01.
